# Keypoint-Aware Single-Stage 3D Object Detector for Autonomous Driving

**DOI:** 10.3390/s22041451

**Published:** 2022-02-14

**Authors:** Wencai Xu, Jie Hu, Ruinan Chen, Yongpeng An, Zongquan Xiong, Han Liu

**Affiliations:** Key Laboratory of Advanced Technology for Automotive Components, Wuhan University of Technology, Wuhan 430070, China; vehfish@whut.edu.cn (W.X.); chenruinan@whut.edu.cn (R.C.); yongpeng20_@whut.edu.cn (Y.A.); zq.xiong@whut.edu.cn (Z.X.); liuhan77@whut.edu.cn (H.L.)

**Keywords:** 3D single stage object detector, feature reuse strategy, location attention module, keypoint-aware module

## Abstract

Current single-stage 3D object detectors often use predefined single points of feature maps to generate confidence scores. However, the point feature not only lacks the boundaries and inner features but also does not establish an explicit association between regression box and confidence scores. In this paper, we present a novel single-stage object detector called keypoint-aware single-stage 3D object detector (KASSD). First, we design a lightweight location attention module (LLM), including feature reuse strategy (FRS) and location attention module (LAM). The FRS can facilitate the flow of spatial information. By considering the location, the LAM adopts weighted feature fusion to obtain efficient multi-level feature representation. To alleviate the inconsistencies mentioned above, we introduce a keypoint-aware module (KAM). The KAM can model spatial relationships and learn rich semantic information by representing the predicted object as a set of keypoints. We conduct experiments on the KITTI dataset. The experimental results show that our method has a competitive performance with 79.74% *AP* on a moderate difficulty level while maintaining 21.8 FPS inference speed.

## 1. Introduction

Nowadays, object detection has become a fundamental task of scene understanding, attracting much attention in various fields, such as autonomous vehicles and robotics. The tasks include traffic sign detection [1,2,3], traffic light detection [4,5], 2D object detection [6], and 3D objection detection [7,8], which rely on sensors installed on the autonomous vehicles. Since LiDAR (light detection and ranging) can provide accurate distance information about the surrounding environment and is not impacted under low-light conditions, it has become one of the main sources of perception. The purpose of 3D object detection of LiDAR point cloud is to predict the bounding box, classification, and direction, an essential job for downstream perception and planning tasks.

Recently, 3D object detection methods based on deep learning have been widely adopted, and achieved dramatic developments in industry and academia [7]. Despite huge advantages, it is important to note that point clouds suffer some drawbacks: (1) The original point cloud is sparse, while the image is dense; (2) Point cloud data have an unstructured and unordered nature [8]; (3) Point cloud data are sensitive to occlusion and distance; (4) 3D features introduce a heavy computational burden. Instead of learning feature for each point, volumetric-based methods encode point clouds into regular 3D grids, called voxels, so as to achieve robust representation and then apply a Convolution Neural Network (CNN) for feature extraction and prediction instance object. Furthermore, a regular data format can naturally transfer previous mature knowledge from the image domain. Although the point cloud can reflect the real geometric structure and object size, the image may suffer from these information losses. Thus, applying image methods directly may deliver the opposite effects and degrade the final performance. Based on the discussion above, this study analyzes the performance of representative architecture on feature extraction. Moreover, we derive a novel and efficient feature extraction method that can learn a rich feature representation and avoid using deeper models that slow the calculation speed.

One of the crucial questions in object detection is the inconsistence between the confidence score and the predicted bounding box. Generally, the confidence score is usually used to rank the bounding box in the Non-Maximum Suppression (NMS) process to remove redundant candidates [9]. It is found that IOU is more responsive to localization quality; thus, 3D IOU-Net [8] and CIA-SSD [10] integrate an IOU prediction head into object detection architecture to achieve a remarkable performance improvement. However, these methods remain problematic in that there is no way to measure the distance between the two bounding boxes when they do not overlap with each other, which does not facilitate the subsequent optimization process.

**Figure 1:**
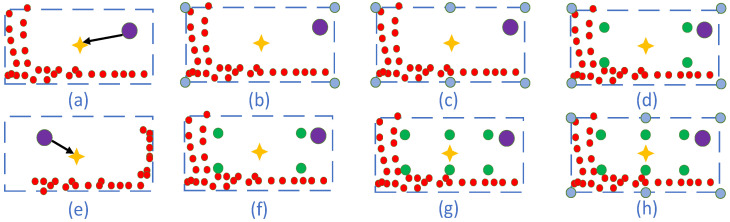
Various BEVs (bird’s eye views) of predicted bounding box. The purple circle refers to the predefined anchor point, which predicts the 3D bounding box. The blue circles refer to the extracted keypoints on the boundary, and the green circles refer to the extracted keypoints inside the bounding box. The blue dashed line refers to the BEV of the predicted bounding box. The red circles refer to the point on the detection object scanned by the LIDAR, the yellow star refers to the center location of the bounding box. (**a**,**e**) denote different relations of predefined anchor points and bounding boxes. (**b**,**c**) denote different distribution of boundary keypoints of the bounding boxes. (**f**,**g**) indicate different distribution of inner keypoints of the bounding boxes. (**d**,**h**) indicate different Point out the different sampling methods covering the boundary and inner parts.

In addition, most 3D object detection methods adopt a predefined single anchor point to calculate object confidence scores. However, in such cases, the location is independent from training and no close ties can be established with the predicted bounding box. Especially, two drawbacks will appear: (1) Although the predefined anchor point position is fixed, the position of the predicted bounding boxes relative to the anchor point is shifted. In Figure 1a, the anchor point (purple circle) stands on the top right of the predicted bounding box, while in Figure 1e, it is on the top left of the predicted bounding box. It can be observed that the relative position of the predefined anchor points to the bounding box is uncertain. In other words, it is necessary to establish an explicit mapping correspondence between the detection object and sampling points; (2) The position of the LIDAR scanning on the object is different due to the position and orientation of the detection object. In Figure 1a, the scan point lies in the lower left part of the bounding box, while in Figure 1e, it is in the lower right part of the bounding box. Obviously, the traditional 3D object detection method using a single anchor point feature cannot adequately describe the whole bounding box feature. Therefore, more robust bounding box representation needs to be explored.

Considering the above problem, we proposed a KAM (keypoint-aware module) which can directly utilize the boundary keypoints from each boundary and inner keypoints by mapping the predicted real geometries to a feature map. In this way, the predicted scores can be jointly optimized with the features corresponding to the prediction bounding boxes.

In the paper, we divide the bounding box into two parts, so as to extract the sampling keypoints: the boundary parts and the inner parts. For the extraction of sampling points, three aspects are considered: (1) The contribution of the boundary points or inner points, respectively, to the 3D object detection; (2) Different sampling strategies for boundary points and inner points. For boundary keypoints sampling, two sampling strategies are adopted. One is to use only the four corner points (blue circles), as in Figure 1b. The other is to sample the boundary uniformly. For example, as seen in Figure 1c, three points are sampled uniformly for the boundary. For inner keypoints sampling, the points are divided into different numbers of uniform spatial grids. For example, in Figure 1f, the BEV of the bounding box is divided into four parts, while in Figure 1g, the BEV of the bounding box is divided into six parts; (3) A combination of boundary keypoints and inner points. The combination of different numbers of boundary and inner keypoints could improve the performance of 3D object detection differently. For example, in Figure 1d, four samples are extracted from the boundary and other four from the inner. In Figure 1h, the boundary parts extract six samples and the inner parts extract six samples. The combination that yields the best detection performance should be chosen.

In summary, the key contributions of the proposed method are as follows:

(1) In order to better retain and extract spatial information from LiDAR, as well as to extract effective cross-layer features, a novel lightweight location attention module named LLM is proposed, which can maintain an efficient flow of spatial information and incorporate multi-level features.
(2) A keypoints sample method is adopted to enhance the correlation between the predicted bounding box and scores, thus improving the performance of detection.
(3) Extensive experiments are conducted on the KITTI benchmark dataset, demonstrating that the proposed network attains good performance.

## 2. Related Work

### 2.1. Multi-Modal Fusion Detector

Currently, the fusion between LiDAR and camera sensors is a promising research hotspot. The camera can provide dense and rich information on objects such as their fine-grained colors and textures, while LiDAR can provide precise range information. Thus, various fusion methods have been exploited to enhance the performance of 3D object detection.

Frustum PointNets [11] assumes the availability of 2D image proposals and gets the point cloud of the corresponding area to estimate the 3D bounding box. However, Frustum PointNets assumes that vertical space only has one object, which is not suitable for the 3D point cloud. Thus, Frustum ConvNet [12] is designed to generate a sequence of proposals to solve the problem of multiple object occlusions. MV3D [13] introduces the two-stage detector. It generates 3D object proposals from the BEV map and deeply fuses multi-view features via region-based representation. Such fusion is with comparative coarse, so DCFS [14] exploits continuous convolutions to fuse image and LIDAR feature maps at different levels of resolution. To guide the network to learn better cross-modality feature representations, MMF [15] develops a multi-task detector, which can deliver 2D and 3D object detection, ground estimation, and depth completion.

In order to avoid forcing fuse feature vectors from different sizes, CLOCs [16] propose a late fusion strategy, which only jointly aligns and label data in the final fusion step.

### 2.2. LiDAR-Based Detector

Judging from the current published work, the performance of LiDAR-only based detectors is better than that of the fusion-based detectors.

There are two approaches to tackle object detection on the point cloud. The first approach is to use a two-stage detector. PointRCNN [17] is such a two-stage detection framework. The first stage generates a foreground segmentation mask, while the second stage conducts region pool operation from the 3D proposals in the first stage, so as to refine 3D bounding boxes. STD [18] designs spherical anchors to generate accurate point-based proposals. To capture dense representations, it deploys a new PointsPool layer to convert point-based features. PV-RCNN [19] incorporates point-based and voxel-based methods to generate high-quality multi-scale features with flexible receptive fields. However, the two-stage methods mentioned above fail to satisfy the real-time requirements. VoxelNet [20] propose new voxel features encoding layer to learn complex feature. SECOND [21] introduces an improved sparse convolution and data augmentation to reduces the time for both training and inference. PointPillars [22] learns features on pillars and significantly increase the speed by formulating all key operations as 2D convolutions. 3DSSD [23] combines F-FPS and D-FPS together to effectively preserve interior points of various instances to solve sampling questions.

### 2.3. Location Quality Estimation

This study is focused on single-stage object detectors, owing to real-time effectiveness. Generally, classification scores are predicted with a single point in a predefined style. SASSD [24] divides feature map corresponding to the bounding box and select the center position to obtain more precise classification scores. However, SA-SSD does not exploit boundary awareness of bounding box, which is also important to define object confidence. 3D IoU-Net [8] use the IOU (Intersection-over-Union) to suppress irrelevant confidence. CIA-SSD [10] formulates a novel IOU-weighted NMS to reduce redundant predictions and keep higher inference speed. However, CIA-SSD and 3D IOU-Net do not associate the predicted bounding box feature with a certain score.

As analyzed above, current advanced methods take into account the confidence scores quality. Different from the previous methods, this study deeply explores the relationship between the confidence scores and the predicted box, so that it can be closely linked to improving detection performance.

## 3. Approach

In this section, we introduce the proposed single-stage 3D object detection method. First, we present the design of full architecture. Second, we discuss the 3D feature extraction backbone module. Third, the proposed location attention module is discussed. Finally, we depict the strategies for keypoint-aware module in detail.

### 3.1. Framework

**Figure 2:**
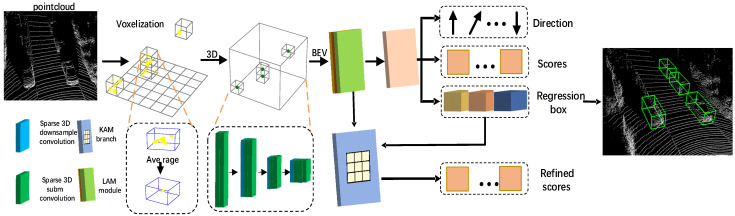
Overview of the KASSD. Firstly, the KASSD convert raw points to voxel features. Then, the 3D backbone module applies 3D sparse convolution for feature extraction. Subsequently, the 3D features are converted into BEV representations, on which we use the LLM module to obtain more expressive features for subsequent detection. Finally, the KAM take regression box is used as input and generates accurate confident scores for post-processing.

The proposed KASSD detector, as depicted in Figure 2, consists of four components: (1) A voxel-based feature extractor; (2) A 3D backbone module; (3) A lightweight location attention module; (4) A keypoint-aware module.

The 3D backbone module voxelizes raw 3D point cloud data and converts these data to 3D sparse CNN features. Then, 3D sparse convolution is used to extract features effectively. Compared to the 3D sparse convolution, 2D convolution is more compact and efficient [25]. Thus, the 3D sparse feature is reshaped to deliver the general 2D feature presentation. The extracted feature information contained in the point cloud is not only in higher sparsity but also substantially varies according to distances. To efficiently learn multi-layer features, a lightweight location attention module is proposed to address this problem. For pixel-wise features, most research uses one point to predict the class scores that are not adequate, thus leading to lower accuracy. By discussing different mapping feature extraction methods with influence on the results, this study proposes a novel keypoint-aware sample module. Experiments show that the novel method based on mapping is an effective way to learn the remote range and sparsity feature.

### 3.2. Voxelization

**Figure 3:**
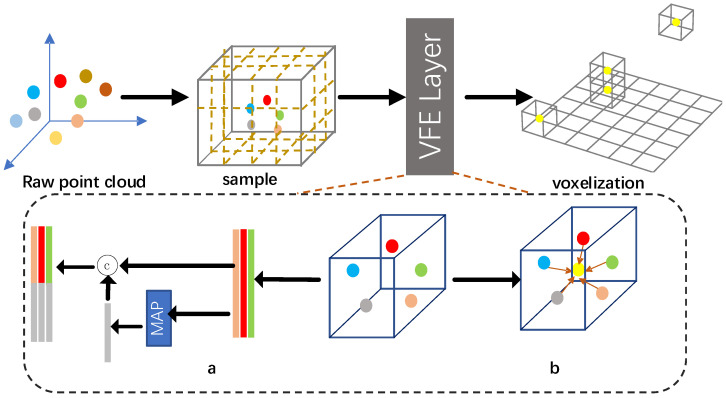
Voxel feature encoding layer. (**a**) Complex encoding method by stacking layers. (**b**) Computing mean value of inner points in voxel grid.

As we all know, the point cloud is unordered and diverse in 3D space; thus, it is necessary that the point cloud is split to heterogeneous small voxels with a resolution of dx, dy, dz, supposing that the range of point cloud is along the X, Y, Z axis. The original point cloud is equally discretized to grid cells with coordinates. Next, it is necessary to make the inter-cell point cloud uniformly. As shown in Figure 3, there exist two methods. Shown in Figure 3a, many papers [26,27] use this method to extract voxel feature, which consists of the FCN layer, pool layer, and feature concatenation layer. It is comparatively complex and consumes some time. In Figure 3b, another method is shown, which is much more simple, and can immediately compute the mean value of all points in a specific grid excluding for empty cell.

### 3.3. D Backbone Module

**Figure 4:**
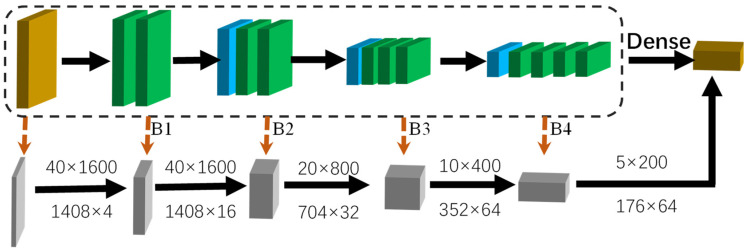
The structure of the 3D backbone module. The first orange box converts voxel features into 4D sparse tensors. The green boxes are submanifold convolutional layers. The blue boxes are sparse convolution with stride = 2.

We use the popular 3D backbone module [28] that consists of four blocks, denoted as $B_{1},B_{2},B_{3},B_{4}$. Each of the blocks is serially connected to the last block. The detailed architecture is shown in Figure 4. Blue layer means submanifold sparse convolutions layer. Green layer means general convolution. Specifically, the input features enter four blocks sequentially, and each one is shown in Figure 4. As a result, these are four stages, where each one has a resolution of with respect to the input feature.

### 3.4. Lightweight Location Attention Module

Aiming to better use the feature representation of point clouds in 2D space with shallow CNN, we introduce the LLA module. Current object detectors mostly use CNN blocks or immediately use multi-level features for feature fusion to strengthen features, which cannot take full advantage of the representation potential of feature fusion. This is because shallow information is obtained through less convolution and lacks rich semantic features, while deep feature lacks excessive spatial detail [29]. However, in the later experiments, we found that the technology which is practical in the imaging field does not scale well in the LIDAR field. Thus, we should exploit a new architecture that can extend the ability of 2D CNN to the point cloud domain.

In the experiments later, we also migrate a large CNN, of which the network depth has also been deepened. In addition, the high-level feature focuses on abstract semantic and provides the rough positions information, while the low-level feature determines the accurate object information. Thus, multi-level feature fusion should consider the importance of space and semantics of different positions in multi-level features.

**Figure 5:**
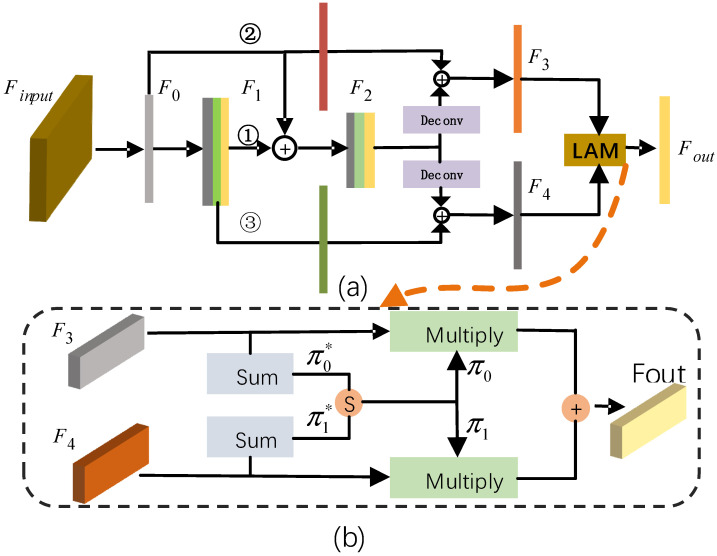
The structure of the LLM module. (**a**) Feature reuse module. (**b**) Location attention module for multi-layer feature fusion.

For spatial information that decreases with increasing network depth, we use a simple but effective method to maintain the efficient flow of spatial information in CNN. The detailed process is shown in Figure 5a. We exploit the potential ability through feature reuse strategy (FRS), yielding representational power from the network.

In Figure 5a, the proposed FRS is mainly implemented in three branches. Firstly, in branch 1, the BEV feature $F_{0}$ is obtained by reducing input feature channels so that they can be directly summed with the following high-level feature. Secondly, the spatial feature $F_{1}$ is obtained by using three convolutions and does not change the feature dimension. Then, the semantic feature $F_{2}$ is obtained by one-layer convolution with stride = 2 and two-layer convolution with stride = 1 to get more abstract semantic representation. Thus, the resolution of $F_{2}$ is reduced by half, but the number of channels doubles. To reduce the loss of spatial information, we add two branches. Branch 2 passes feature $F_{0}$ to each of the subsequent layers. In branch 3, the intermediate layer feature $F_{3}$ also directly passes to the subsequent feature map. $F_{2}$ is reshaped to the original dimension by two deconvolutions so that it can be easily operated with the original feature. In other words, one branch combines the bottom layer feature, which promotes the flow of spatial information, while another branch combines the intermediate feature, which extracts rich semantic features. In addition, we combine features through sum operations. The aim is to reduce the number of parameters; it has been found that this works rather robustly in our experiments. In this way, all features are utilized multiple times.

For the sake of better incorporating multi-level features, we introduce the location attention module (LAM). Generally, the more parameters, the more difficult the training, and this will reduce the speed of inference. Different from AttaNet [29], our module does not introduce any convolution operations, and therefore, reduces the number of parameters. In Figure 5b, the architecture of the SSA module is illustrated in detail.

(1) $$\begin{array}{c} \pi_{0}^{*}=\varphi (F_{3})\\ \pi_{1}^{*}=\varphi (F_{4})\\ \pi_{0},\pi_{1}=softmax(\pi_{0}^{*},\pi_{1}^{*}) \end{array}$$

where φ means element summation operation of feature map, softmax means the SoftMax function, and $\pi_{0},\pi_{1}$ is the attention map of the input features $F_{3}$ and $F_{4}$, respectively.

The input feature of the LAM module consists of a high-level feature $F_{3}\in R^{h\times w\times c}$ and a low-level feature $F_{4}\in R^{h\times w\times c}$ corresponding to parts in Figure 5b. First, in Equation (1), by φ operation, which means element summation, we reduce the feature channel to one by adding values along channel dimension, getting a feature map feature map $\pi_{0}^{*}\in R^{h\times w\times 1}$ and $\pi_{1}^{*}\in R^{h\times w\times 1}$. Next, we use the SoftMax operation to calculate relative attention mask between multi-level feature map which output two BEV attention maps, $\pi_{0},\pi_{1}$. We can view the attention mask as an important weight distribution for each element in the feature. The higher the scores, the more important the position. Finally, since the dimension of the attention map $\pi_{0},\pi_{1}$ is the same as the input feature $F_{3}$ and $F_{4}$, except for the channel dimension, we multiply the input feature by attention map directly. In Equation (2), our adaptive weighted result is calculated as follows:

(2) $$F_{output}=\pi_{0}•F_{3}+\pi_{1}•F_{4}$$

The final output feature map $F_{output}$ is fed into the KAM module for the object detection task.

### 3.5. Keypoint-Aware Module

The purpose of this module is to make full use of the feature information provided by the predicted bounding box. Traditional methods use a single point to represent the proposal, ignoring geometric information and internal feature clues of the entire bounding box.

Most of the point clouds are located on the boundary of the object, which indicates that boundary features are discriminative to the object. In addition, internal features are also essential to the representation of the bounding box, which provides the abstract semantic feature of the object. However, extracting features from the entire region increases the computational burden. Inspired by the R-fcn [30], we devise an effective proposal feature extraction method.

**Figure 6:**
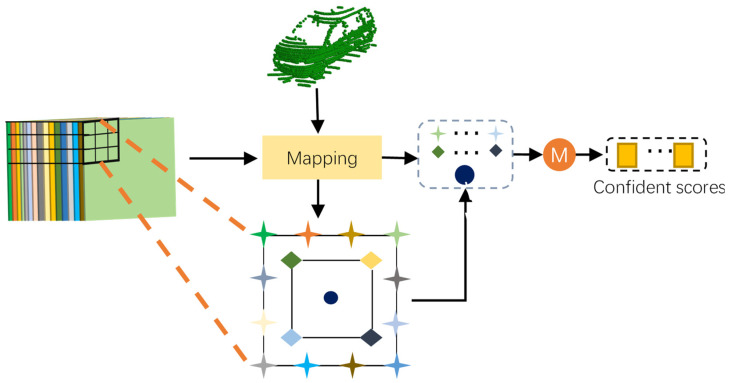
The illustration of the KAM module. The predicted bounding box is projected to feature map and extract keypoints to yield a rich representation.

Thus, we introduce a keypoint-aware module (KAM) for the score prediction. The structure of KAM is shown in detail in Figure 6. To generate effective feature representation for each prediction box in the current training process, the KAM module uses the features at the boundary (star) and inner area (diamond and central circle) sampling points to represent the bounding box. It can capture rich semantic features of the whole object and establish explicit location-feature map relation, which is essential to alleviate the misalignment problem between scores and the prediction bounding box. Specifically, we divide the object detection scores into two parts: a boundary-aware module and an inner-aware module.

The KAM module takes the last layer feature map as the input and consists of three convolutions to output the confident map.

**Figure 7:**
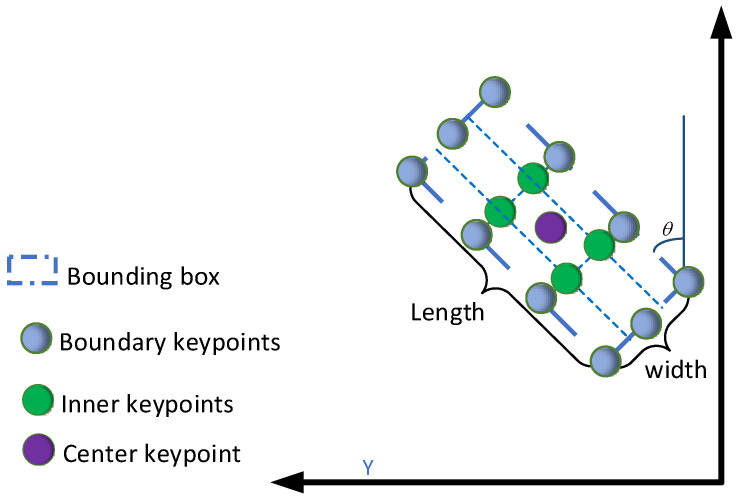
The method to calculate keypoints. The boundary keypoints are represented by blue circles and the inner points by green circles. The center keypoint is indicated by a purple circle. The blue dashed line refers to the BEV of the predicted bounding box.

To the boundary area, our boundary-aware module selects representative features using uniform sampling, such as the blue circle in Figure 7. Specifically, long edges are represented by m points and short edges by n points. Since the labeled boxes are rectangular, the number of keypoints on opposite sides is the same. Given the proposal bounding box from the regression branch, subscript i indicates the i-th point along the *x*-axis and subscript j indicates the j-th point along the *y*-axis. $\left(x_{0},y_{0}\right)$ is the center location coordinate of the bounding box, represented by the purple circle in Figure 7. Lastly, w,l,θ are the width, length, and angle.

The above values are in the LiDAR coordinate system. Moreover, the boundary keypoints calculation method can be defined as follows:

(3) $$k_{i}=\left\{ \begin{array}{l} (x_{0}+\frac{l}{2}\cos(\theta )+n_{i}w\sin(\theta ),y_{0}+\frac{l}{2}\sin(\theta )+n_{i}w\cos(\theta )),E_{l}\\ (x_{0}-\frac{l}{2}\cos(\theta )+n_{i}w\sin(\theta ),y_{0}-\frac{l}{2}\sin(\theta )+n_{i}w\cos(\theta )),E_{r}\\ (x_{0}-\frac{w}{2}\sin(\theta )+m_{i}l\cos(\theta ),y_{0}+\frac{w}{2}\sin(\theta )+m_{i}l\sin(\theta )),E_{b}\\ (x_{0}+\frac{w}{2}\sin(\theta )+m_{i}l\cos(\theta ),y_{0}-\frac{w}{2}\sin(\theta )+m_{i}l\sin(\theta )),E_{t} \end{array}\right.$$

where $n_{i},m_{i}$ are a set of keypoints linearly spaced between $\left[-\frac{1}{2},\frac{1}{2}\right]$. In Figure 7, for example, the long edge is represented by four keypoints, $n_{i}=[-\frac{1}{2},-\frac{1}{6},\frac{1}{6},\frac{1}{2}]$. Moreover, the wide edge is represented by three keypoints, $n_{i}=[-\frac{1}{2},0,-\frac{1}{2}]$. Lastly, $E_{l},E_{r},E_{b}$, and $E_{t}$ denote the left, right, bottom, and top edges, respectively.

Given a certain area inside a bounding box, the inner-aware module divides the inner area evenly into the d×e grid, using grid vertices as feature points. In addition, the calculation method is similar to the boundary points.

If the proposed box is represented by a total of K keypoints, then the corresponding final convolution outputs the K-layer feature map on the so-called score map. Each score map describes the feature response for keypoints of the predicted bounding box. For example, the first scores map represents the score of the top-left point. Assuming the input feature maps are in the order of (left border, top border, right border and bottom border, inner point), each feature point score *F* can be formulated with the following equation:

(4) $$F_{i}(\tau_{x},\tau_{y})=\{\begin{array}{l} \zeta (\rho (K_{i}^{l})),0\leq c<n\\ \zeta (\rho (K_{i}^{r})),n\leq c<2n\\ \zeta (\rho (K_{i}^{b})),2n<c\leq 2n+m\\ \zeta (\rho (K_{i}^{t})),2n+m<c\leq 2n+2m\\ \zeta (\rho (K_{i}^{c})),2n+2m<c\leq 2n+2m+f \end{array}$$

where f denotes the total number of inner points. f=(d−1)×(e−1)+1. $\left(\tau_{x},\tau_{y}\right)$ is a uniform representation for mapped coordinates of the keypoint. $K_{i}$ represents real-world coordinates; moreover, $K_{i}=(x,y)$. *x*, *y* are the coordinates with respect to the LiDAR coordinate system. Typically, $K_{i}$ is fractional; thus, the $F_{i}(\tau_{x},\tau_{y})$ value is calculated by bilinear interpolation ζ with the adjacent position. ρ signifies the coordinates offset and scale, $\rho =\frac{x+offset}{scale}$. $\left(\tau_{x},\tau_{y}\right)$ are the coordinates with respect to the feature map. The mean value of all keypoints is calculated as final scores.

(5) $$s_{i,j}=\frac{1}{N}\sum_{i=1}^{N}F(\tau_{x},\tau_{y})$$

### 3.6. Loss Function

The loss function of our work is a combination of the Regression loss function, class loss function, direction loss function, and keypoints loss function. For the bounding box, the parameterizations of seven coordinates are employed, as in [21]:

(6) $$\begin{array}{l} t_{x}=\frac{x-a_{x}}{a_{d}},t_{x}^{*}=\frac{x^{*}-a_{x}}{a_{d}},t_{y}=\frac{x-a_{y}}{a_{d}},t_{y}^{*}=\frac{x^{*}-a_{y}}{a_{d}}\\ t_{z}=\frac{x-a_{z}}{a_{h}},t_{x}^{*}=\frac{x^{*}-a_{z}}{a_{h}},t_{w}=log(\frac{w}{a_{w}}),t_{w}^{*}=log(\frac{w^{*}}{a_{w}})\\ t_{l}=log(\frac{l}{a_{l}}),t_{l}^{*}=log(\frac{l^{*}}{a_{l}}),t_{z}=log(\frac{h}{a_{h}}),t_{z}^{*}=log(\frac{h^{*}}{a_{h}})\\ t_{\theta }=g_{\theta }-a_{\theta },t_{\theta }^{*}=g_{\theta }^{*}-a_{\theta } \end{array}$$

where $a_{d}=w_{a}^{2}+w_{b}^{2}$. The box’s center coordinates and its width, length, height, and angle are, respectively, denoted as x,y,z,w,l,h,θ. Parameters $x,x^{*}$ and $a_{x}$ are for the predicted box, ground truth box, and anchor box, respectively. Likewise, y,z,w,l,h,θ.

One-stage 3D object detection faces an extreme imbalance during training. Thus, we commonly use focal loss [31] to deal with this problem by assigning well-classified examples with lower weights:

(7) $$FL\left(p_{i}\right)=-\alpha {\left(1-p_{i}\right)}^{r}log(p_{i})$$

For box regression, $Smooth_{L1}$ loss is adopted, as shown as follows:

(8) $$Smooth_{L1}=\{\begin{array}{l} 0.5x^{2},\left|x\right|<1\\ \left|x\right|-0.5,otherwise \end{array}$$

Thus, the box loss can be defined as:

(9) $$L_{loc}=\frac{1}{N_{pos}}\sum_{i\in Pos}^{N}\sum_{m\in x,y,z,w,l,h,\theta }Smooth_{L1}(t-t*)$$

where $t,t^{*}$ indicate the predicted encoded value and ground truth encoded value in Formula (1), respectively.

We use the discrete approach by introducing sin function $sin(t_{\theta }-t_{\theta }^{*})$ and direction classifier cross-entropy.

Hence, the multi-task loss function of our KASSD for end-to-end training is calculated as follows:

(10) $$L_{total}=\lambda_{1}L_{cls}+\lambda_{2}L_{loc}+\lambda_{3}L_{kp}+\lambda_{4}L_{dir}$$

where $\lambda_{1},\lambda_{2},\lambda_{3},\lambda_{4}$ are hyper-parameters that weight each loss term of multi-task learning.

## 4. Experiments

### 4.1. Dataset and Evaluation Metrics

We comprehensively conduct experiments on the KITTI dataset [32], a large-scale dataset for LiDAR point-cloud object detection. The dataset contains 7481 training samples and 7518 test samples. Following previous work [13,26,33] the training samples are split into a training set consisting of 3712 samples and a validation set consisting of 3769 samples, which is about a 1:1 ratio.

Furthermore, the KITTI datasets have three levels: easy, moderate, and hard, which depend on the size, occlusion, and truncation levels. To facilitate comparison with previous work, we use the car category and calculate the average precision (*AP*) to evaluate the result for different difficulty levels.

*AP* is the common evaluation metric for 3D object detection in the KITTI. Specifically, the *AP* summaries the shape of the precision/recall curve, and is defined as the mean precision at a set of equally spaced recall levels. In KITTI, 41 recall positions are official evaluation protocol. For example, $AP_{11}$ is calculated according to the following equation:

(11) $$AP=\frac{1}{11}\int_{0}^{1}p(r)dr$$

where r is a set of 11 recall values linearly spaced between [0, 1].

Following the metrics used in the previous paper, we calculate *AP* with 11 recall positions to evaluate the validation set.

In addition, practical application of research is one of the important criteria for judging work. Thus, we establish a dataset of 3D object detection for our school scene named the WHUT dataset. Our dataset contains 2000 frames of samples, annotated over one month. Furthermore, we use 1500 frames for training and 500 frames for validation. Moreover, we adopt the same metrics as KITTI without difficulty division.

### 4.2. Implementation Details

We use the most commonly voxelization with grid of [0.05, 0.05, 0.1] meters and crop the original point cloud in ranges [0, 70.4], [−40, 40], [−3, 1] meters along the x, y, z axes. Every point on the last layer of feature corresponds to two pre-defined boxes, which have the same size (width = 1.6 m, length = 3.9 m, height = 1.56 m) and different orientations (0° and 90°).

The network is trained by Adaptive Moment Estimation (Adam) [34] with the cosine annealing learning rate. In addition, our model is trained for 80 epochs with a batch size of two on four 2080Ti GPU cards. In the experiments, we set $\lambda_{1},\lambda_{2},\lambda_{3},\lambda_{4}$ to 2, 1, 1, and 0.2, respectively.

### 4.3. Evaluation with the KITTI Dataset

We evaluate the performance of our KASSD with the KITTI dataset by submitting the detection results to the KITTI server for evaluation. As shown in Table 1, our proposed method outperforms most existing methods, such as 3DSSD, DVFENet, SECOND, STD, PointRCNN, and TANet, by roughly 0.04 to 3.0 points, but is slightly inferior to 3D-SSD on hard *AP*. The performance of KASSD in the validation set of the car category is shown in Table 2.

**Figure 8:**
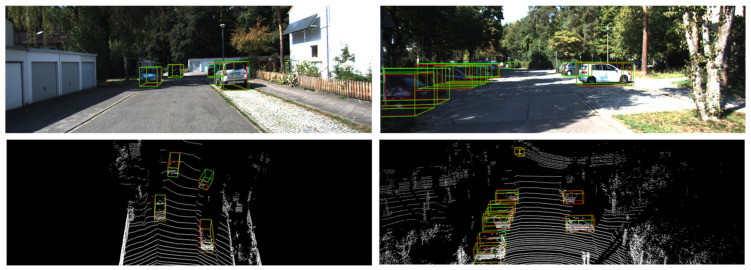
A visualization result of cars using the KITTI validation set. We present paired samples, where in each pair, row 1 is the 3D bounding boxes projected into the image for clearer visualization, while row 2 is the detection result of the LiDAR point cloud. We use red and green boxes to denote detections and ground truth boxes, respectively.

**Figure 9:**
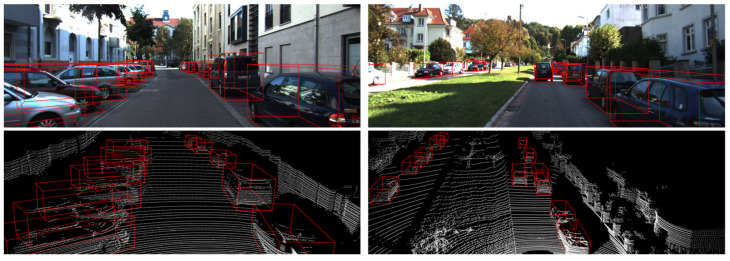
A visualization result of cars using the KITTI test set. The detection results are indicated by a red box.

From the results, it can be observed that our method outperforms several other methods across all difficulty levels. Our method performance outperforms other methods on moderate and hard levels. The lack of labeling on the distant and heavily occluded object is the primary difference in cause. In some cases, despite the fact that the network learns features, the object detected in certain situations is deemed to be incorrect. Another cause is the dataset’s inconsistent distribution, as mentioned in CIA-SSD [10]. In addition, 3D IoU-Net [8] uses a single point to predict IOU, whereas our approach employs richer regional information to increase the network’s ability to estimate the confidence scores of the bounding box. In Table 1, it is obvious that our method achieves better performance. We also show some qualitative result of validation and test sets in Figure 8 and Figure 9, respectively.

### 4.4. Evaluation on the WHUT Dataset

We show the performance of our model and SECOND in Table 3 and compare their *AP*. In addition, the WHUT dataset is recorded by 32-beam LiDAR, which has a lower resolution than the 64-beam LiDAR utilized by KITTI. However, our model still outperforms SECOND 0.97 *AP*. It demonstrates that our proposed KASSD object detector is still effective in low-resolution LiDAR.

### 4.5. Ablation Study

To demonstrate the effectiveness of the proposed module, we compare the proposed KASSD to a baseline detector. Table 4 presents the results of *AP* with algorithms equipped with various submodules. First, we investigate the effect of LLM by substituting the proposed module for the convolution component. Compared with the baseline, easy, moderate, and hard *AP* are 0.32, 0.4, and 0.27 higher than the baseline, respectively, demonstrating that the LLM module better retains spatial information and fuses high-resolution and sematic features to concentrate more on discriminative information. We further conduct experiments on KAM. The validation results show that the proposed module significantly outperforms the baseline methods with 0.85, 0.89, and 1.21 on all difficulty levels. Moreover, our proposed method outperforms baseline model by 1.05, 1.11, and 1.24, especially when all submodules are combined.

To further highlight the performance of the LLM relative to other feature extraction modules, we compare it with other advanced approaches in Table 5. TANet [33] is a new feature fusion method that has been proposed recently, using pyramid modules. Moreover, we replaced the LLM modules with PSA modules for training. The result shows that the PSA improves the *AP* significantly on the easy level. However, for the moderate and hard levels, the *AP* value drops over 0.3. This is proof that our method has more powerful spatial and semantic information extraction capabilities compared to other advanced methods.

We also exploit the impact of the receptive field to verify the suitability of traditional enhancement techniques in point clouds. The astrous convolution is an effective method for enlarging the receptive field and play an important role in object detection. Thus, we insert SPP [45] and DENSEASPP [46] separately into the intermediate convolution layer to test the effect of receptive field. We can find that the *AP* in each difficulty level drops dramatically. In other words, a direct increase in the receptive field using astrous convolution does not contribute to the performance, which also validates the difference between sparse point clouds and dense image features.

In Table 6, we delve into the representational power of various parts of the bounding box and different numbers of sampling points. Firstly, we analyze the effect of the four boundary corner points, which enhance the easy, moderate, and hard *AP* by 0.33, 0.35, and 0.48, respectively. In addition, experiments on the increase of boundary points were also conducted. The detection performance was improved again when the number of boundary points was increased to 18; the easy, moderate, and hard *AP* was increased by 0.23, 0.15, and 0.16. However, when the number of boundary points was increased to 28, the easy and moderate *AP* dropped slightly. Based on the results of the proceeding experiments, it is clear that the representation capability of the predicted bounding box is improved by increasing the number of keypoints.

Next, we examine the best performance in the case of using inner keypoints. We divide the internal area evenly into grids and take joints as keypoints for sampling. Following the previous convention of the score, we still add predefined anchor points as one of the sampling points. As a result, we were able to obtain 11 keypoints. For the inner points, we observe a huge *AP* improvement of 0.8, 0.81, and 0.78 for easy, moderate, and hard levels, demonstrating the importance of describing the inner area for an object representation method.

Table 6 further shows how employing a variety of sampling points over the entire area improves the *AP*. We combine the sampling of the inner keypoints with the boundary keypoints and call it mixed keypoints sampling. Compared to boundary keypoints (Row 3), mixed keypoints sampling outperforms it by 0.29, 0.39, and 0.51 *AP* on the easy, moderate, and difficult levels. To inner keypoints (Row 5), mixed keypoints sampling brings an improvement of 0.37 on the hard level of difficulty. This indicates that mixed keypoints sampling achieves higher performance than sampling with only boundary or internal key-points. Thus, we are able to conclude that boundary keypoints and inner keypoints can complement each other to improve the performance of 3D object detector.

In addition, we note that varying the number of keypoints sampling has an impact on the results. In Row 7, we can find that performance degrades when too many mixed keypoints sampling are extracted.

### 4.6. Runtime Analysis

Running speed, particularly in autonomous driving, plays an important role in object detection. Furthermore, the speed of inference fluctuates in a small range. Thus, all runtimes were averaged from ten runs of the algorithm.

The average inference time of our method is 45.9 ms. The inference time is calculated as follows. The point cloud must first be loaded and preprocessed (2.3 ms). The data tensor is then processed by KASSD (42.9 ms). Moreover, post-processing was done to get the final result (0.7 ms). Because real-time detection is critical in autonomous driving, we examine the detection speeds of several approaches. In Table 7, we can find that our proposed KASSD is 597.1 ms, 14.1 ms, and 0.4 ms faster than point-GNN, Associate-3Ddet, and SECOND, respectively. Compared with 3DSSD, the inference speed of our model is slightly slower. However, our model outperforms 3DSSD by 0.43, 0.89, and 0.24 *AP* on easy, moderate, and hard levels of difficulty, respectively. In other words, KASSD improves the 3D object detection performance with tolerable computation overhead.

### 4.7. Discussion

We proposed a simple keypoint-aware module for 3D object detection, which has four advantages. Firstly, our proposed KAM (keypoint-aware module) solves the problem that the relative position of the predefined anchor point and predicted bounding box is uncertain in the traditional 3D single-stage object detection. Secondly, experimental results show that both boundary and inner points can improve the performance of the 3D object detector. This also illustrates that the predefined anchor points of conventional 3D object detectors lack sufficient information, which may lead to a decrease in performance. Thirdly, the ideal option is to use sampling of a mix of keypoints. It indicates that inner keypoints and boundary keypoints adequately capture the context of the predicted bounding box and effectively produce high-quality object description. Finally, our proposed method achieves competitive performance.

However, there are some limits to our method. Our proposed method uses a fixed keypoints sampling mechanism for all predicted objects and cannot adaptively select the best keypoints. It may impede the ability of the 3D object detectors to perform well. In the future, we will focus on a learnable keypoint-aware module, which may result in more significant improvements.

## 5. Conclusions

This paper presents a novel KASSD for one-stage object detection in point clouds. The feature reuse strategy (FRS) and location attention module (LAM) maintain spatial information flowing smoothly and extract representative features in order to predict an accurate 3D bounding box. Then, we reveal the limitations of traditional single-stage 3D object detection and proposed the Keypoint-Aware Module (KAM), which projects the 3D bounding box to the feature map of the corresponding channel to adequately capture the context of the predicted bounding box. Experimental results on the KITTI dataset demonstrate that our method outperforms many 3D object detection methods on KITTI benchmark, which suggests that the proposed method is suitable for 3D object detection in the point cloud. The learnable keypoint-aware module, which can adaptively select the location and determine the placement of keypoints to further improve the performance of the 3D object detector, will be the focus of a future study.

## Figures and Tables

**Table 1:** Results of the KITTI test set using the car category.

Type	Method	Modality	*AP*		
			Easy	Mod	Hard
2- stage	MV3D [13]	LiDAR + Camera	74.97	63.63	54.00
	F-PointNet [11]	LiDAR + Camera	82.19	69.79	60.59
	PI-RCNN [35]	LiDAR + Camera	84.37	74.82	70.03
	PointRCNN [8]	LiDAR	85.94	75.76	68.32
	Fast Point RCNN [36]	LiDAR	84.28	75.73	67.39
	STD [18]	LiDAR	87.95	79.71	75.09
	VoxelNet [20]	LiDAR	77.49	65.11	62.85
	DVFENet [37]	LiDAR	86.20	79.18	74.58
	3D IoU-Net [8]	LiDAR	87.96	79.03	72.78
1- stage	SECOND [21]	LiDAR	87.44	79.46	73.97
	PointPillars [22]	LiDAR	82.58	74.31	68.99
	TANet [33]	LiDAR	84.39	75.94	68.82
	Associate-3Ddet [38]	LiDAR	85.99	77.40	70.53
	Point-GNN [39]	LiDAR	88.33	79.47	72.29
	3DSSD [23]	LiDAR	88.36	79.57	74.55
	HCNET [40]	LiDAR	81.31	73.56	68.42
	AVEF [41]	LiDAR	84.41	75.39	69.89
	Ours	LiDAR	88.92	79.75	72.17

**Table 2:** Results of the KITTI validation set using the car category.

Type	Method	Modality	*AP*		
			Easy	Mod	Hard
2- stage	MV3D [13]	LiDAR + Camera	86.55	78.10	76.67
	F-PointNet [11]	LiDAR + Camera	88.16	84.02	76.44
	PI-RCNN [35]	LiDAR + Camera	88.27	78.53	77.75
	PointRCNN [8]	LiDAR	88.88	78.63	77.38
	Fast Point RCNN [36]	LiDAR	89.12	79.00	77.48
	STD [18]	LiDAR	89.7	79.8	79.30
	VoxelNet [20]	LiDAR	81.97	65.46	62.85
	DVFENet [37]	LiDAR	89.81	79.52	78.35
	3D IoU-Net [8]	LiDAR	89.31	79.26	78.68
1- stage	SECOND [21]	LiDAR	87.43	76.48	69.10
	PointPillars [22]	LiDAR	88.91	79.88	78.37
	TANet [33]	LiDAR	87.52	76.64	73.86
	CIA-SSD [10]	LiDAR	90.04	79.81	78.80
	Associate-3Ddet [38]	LiDAR	89.29	79.17	77.76
	Point-GNN [39]	LiDAR	87.89	78.34	77.38
	3DSSD [23]	LiDAR	89.71	79.45	78.67
	HCNET [40]	LiDAR	88.45	78.01	77.72
	EPGNet [42]	LiDAR	89.30	78.98	77.79
	AVEF [41]	LiDAR	87.94	77.74	76.39
	PSANet [43]	LiDAR	89.02	78.70	77.57
	RAVD [44]	LiDAR	89.61	79.04	77.81
	Ours	LiDAR	90.14	80.06	78.91

**Table 3:** Results on the WHUT dataset using the car category.

Method	Modality	*AP*
SECOND	LiDAR	72.31
Ours	LiDAR	73.28

**Table 4:** *AP* of different module settings.

LLM	Keypoints	*AP*		
		Easy	Mod	Hard
		89.09	78.95	77.67
√		89.41	79.35	77.94
	√	89.94	79.84	78.82
√	√	90.14	80.06	78.91

**Table 5:** *AP* of different feature extraction modules.

Module	*AP*		
	Easy	Mod	Hard
PSA	90.14	79.61	78.35
DENSEASPP	89.74	79.12	78.08
SPP	89.62	79.42	78.32
Ours	90.14	80.06	78.91

**Table 6:** *AP* of different keypoint settings.

Boundary Points	Center Points	*AP*		
		Easy	Mod	Hard
0	0	89.09	78.95	77.67
4	0	89.42	79.30	78.15
18	0	89.65	79.45	78.31
28	0	89.62	79.42	78.32
0	11	89.89	79.76	78.45
18	13	89.94	79.84	78. 82
28	19	89.66	79.32	78.43

**Table 7:** *AP* of different keypoint settings.

Method	Point-GNN	Associat-3Ddet	Second	3DSSD	Ours
Speed (ms)	643	60	46.3	38	45.9

## Data Availability

Publicly available datasets were analyzed in this study. These data can be found here: http://www.cvlibs.net/datasets/kitti/eval_3dobject.php (accessed on 18 May 2021).
